# The Ethic of Responsibility: Max Weber’s *Verstehen* and Shared Decision-Making in Patient-Centred Care

**DOI:** 10.1007/s10912-019-09577-7

**Published:** 2019-12-06

**Authors:** Ariane Hanemaayer

**Affiliations:** 1 Department of Sociology, Brandon University, 270 18th Street, Brandon, MB R7A 6A9 Canada; 2 Centre for Research in Arts, Social Sciences and Humanities, University of Cambridge, 7 West Road, Cambridge, CB3 9DT UK

**Keywords:** Max Weber, Verstehen, Shared decision-making, Patient-centred care, Evidence-based medicine

## Abstract

Whereas evidence-based medicine (EBM) encourages the translation of medical research into decision-making through clinical practice guidelines (CPGs), patient-centred care (PCC) aims to integrate patient values through shared decision-making. In order to successfully integrate EBM and PCC, I propose a method of orienting physician decision-making to overcome the different obligations set out by a formally-rational EBM and substantively-rational ethics of care. I engage with Weber’s concepts “the ethic of responsibility” and *verstehen* as a new model of clinical reasoning that reformulates the relationship between medical knowledge and social values, while demonstrating the relevance of the classical sociological cannon to contemporary medical humanities.

Consider modern medicine, a practical technology which is highly developed scientifically. The general “presupposition” of the medical enterprise is stated trivially in the assertion that medical science has the task of maintaining life as such and of diminishing suffering as such to the greatest possible degree. Yet this is problematical. . . . Whether life is worthwhile living and when – this question is not asked by medicine. Natural science gives us an answer to the question of what we must do if we wish to master life technically. It leaves quite aside, or assumes for its purposes, whether we should and do wish to master life technically and whether it ultimately makes sense to do so. (Weber [1919] 1946, 144)

Max Weber’s well-known comments about the separation between science and values are helpful for thinking about the ethical dilemmas that have emerged as a result of efforts to integrate evidence-based medicine (EBM) with patient-centred care (PCC). EBM is defined as the conscientious and judicious use of medical research (“evidence”) in clinical practice (Sackett et al. 2000, 1). Under EBM, the best evidence is considered to be the results of randomized controlled trials (RCTs) (Guyatt et al. 2008). RCTs measure the effectiveness of therapies and medical intervention. The results are subjected to statistical modeling in order to demonstrate the usefulness of a therapy so that a doctor can apply this knowledge in her/his/their practice. EBM aims to master the practice of medicine through the use of evidence to improve medical decision-making.

This paper aims to synthesize EBM with PCC through Max Weber’s political sociology, which provides a method to integrate facts and values in decision-making. To make my argument, first I introduce an exemplary case that highlights the conflict between medical evidence/facts and patient values. Next, I define the nature of evidence in shared decision-making as one way to integrate EBM with PCC. I demonstrate that these views aim to improve the sharing of medical facts yet fail to capture patient values. In order to provide a method that integrates these two approaches, I explain the nature of the conflict by drawing on the underlying forms of rationality that guides medicine’s attempts to systematize the uptake of evidence (clinical practice guidelines) and values (codes of ethics). Both of these rationalities, I argue, are too abstract and render decision-making a technical procedure that is guided by general rules. Weber is helpful for resolving the dilemmas of rule-following and the fact/value distinction because those who occupy leadership roles, such as doctors who are entrusted with managing our health, can act in nontechnical ways. That is, if doctors are guided by a sense of responsibility, or, as I argue, in the case of medicine, a shared responsibility, patient values can be integrated into decision-making in ways that are guided by patient values. For PCC to be successfully integrated with EBM, decision-making requires a method that is not formalized to rules but is attentive to the messiness of the clinic and seeks to overcome the conflict through a patient-oriented method of decision-making, which is not rule-guided.

## Decision-making in medicine: the conflict between EBM and PCC

Making decisions is far from simple in the clinic. For example, in a Sunrise Rounds blog post titled, “Against Medical Advice?” on August 3, 2012, Doctor James Salwitz, an oncologist, writes about a recent difficult decision:

Stan is a 57-year-old man with curable colon cancer who requires surgery. Unfortunately, that surgery will result in a colostomy.^1^ Without that specific operation, there is an increased risk the cancer will spread. Stan is smart, aggressive and independent. He wants us to modify the treatment to avoid the colostomy. However, such a compromise is outside standard of care, and not supported by what modern medicine understands about colon cancer treatment.

In my office, Stan and I talk at length and he poses a challenging question. He says, “Doc, if I was a friend of yours what would you recommend?” After a moment of thought, I decide that is easy. I would give a recommendation that makes cure most likely. Hundreds of thousands of patients live active lives with colostomies, but very few live such lives with active colon cancer. Therefore, I would tell my friend to have the surgery, accept the colonoscopy, giving the most compassionate support I could muster.

However, then I had a conversation inside my own head. I asked me, Jim Salwitz, not Dr Salwitz, what I would do... To my surprise, I had a moment of bright light insight and intuition. I might very well accept the risk of colon cancer recurrence, nary even death, and not get the colostomy. I was stunned! Patient Jim overrides Dr Jim...

This anecdote points to the fact that despite the best evidence, the doctor is confronted with an ethical dilemma about what to recommend to his patient about the operation. If Salwitz was following EBM, he would make decisions based on the best possible outcome, which he knows from medical research is the operation. But the quality of life and the patient’s values come into conflict with this determination. The doctor is conflicted about what he ought to do.

Critics of EBM have said that the use of guidelines in medical practice raises questions about ethics – what the doctor *ought* to do with the evidence, and how she/he/they ought to interpret these general rules into their practices. For example, in an analysis essay published in the *British Journal of Medicine*, the evidence-based Renaissance Group stated that EBM is now a movement in crisis. EBM was defined as a movement that changed medicine (Greenhalgh, Howick, and Maskery 2014). Now, however, medicine is no longer true to the principles of EBM: there is an “overemphasis on algorithmic rules” (19). True evidence-based practice, the authors state, is not bound by rules but relies on a strong relationship between physicians and patients (20). The EBM model, however, has been criticized for its lack of patient-centredness (e.g., Mead and Bower 2003, 1103).

Patient-centred care (PCC) is defined as “the need for clinicians, staff, and health care systems to shift focus away from diseases and back to the patient and family” (Barry and Edgman-Levitan 2012, 780). Various attempts have been made to synthesize the values of medicine with the values of the patient and his/her/their family. Evidence-based patient-centred care is defined as “the use of evidence-based information as a way of enhancing people’s choices when those people are patients” (Elwyn and Edwards 2009, 7). The concern in the PCC literature, however, is that evidence under EBM can be at odds with patient experience and values because patient experience is difficult to quantify and is thus unreliable and irrational under EBM (Cronje and Fullan 2003, 357-358). Under EBM, physicians are expected to use their clinical expertise to integrate the best evidence, but critics have shown that not only is there a tension between the evidence and evidence-based guidelines and clinical expertise (e.g., Greenhalgh 1999, 323), but “the status of scientific data as evidence rests not only in the research itself, but in the diverse communities of physicians who interpret and use them” (Berkwits 1998, 1542). Further, the value of the facts derived from medicine about the patients’ experience becomes meaningful within their communities and in relation to cultural values. The integration of EBM with PCC requires a way to move from evidence to practice, or facts to decision, that respects the social elements of the clinical encounter. PCC emphasizes the patient’s autonomy and role within decision-making, yet under EBM, there is a conflict between patient autonomy and the doctor’s need to make good judgments (Godophin 2009, e188).

The PCC approach aims to overcome these issues. Recently, PCC has found its way into the EBM literature through the method of shared decision-making (SDM), where “both parties share information: the clinician offers options and describes their risks and benefits, and the patient expresses his or her preferences and values” (Barry and Edgman-Levitan 2012, 781). One of the challenges in clinical practice that SDM aims to overcome is the requirement to give patients informed choice. Patients, however, come to the clinic with information that lies beyond the evidence-base. For example, they may have done their own online research, or they may have obligations and values that guide their decisions. In the literature, SDM has been primarily focused on the creation of decision-aids for physicians to share with their patients: “Good shared decision making requires clinicians to have access to detailed knowledge and ideally summaries of the latest evidence and the means to share it in a way that supports thoughtful deliberation” (Agoritsas et al. 2015). These aids come in a variety of formats, including a move towards electronic formats (e.g., Agoritsas et al. 2015). The aids aim to provide patients with information to help them make decisions consistent with their values and based on medical knowledge and perception of risk in order to reduce ambivalences and increase patient involvement in their own care (Barry and Edgman-Levitan 2012, 781). Research in the social sciences can help improve SDM models (e.g., Statterfield et al. 2009). If PCC and EBM combine, there can be a conscientious and judicious search for choices that “respond to patient’s ideas, concerns and expectations” (Godophin 2009, e187). SDM allows each to learn from the other and facilitates shared responsibility in any decision about how to proceed.

There remains debate, however, in the literature about how to implement SDM in practice. In some cases shared decision-aids are recommended (e.g., Giguere et al. 2014). In other cases, better forms of communication are suggested (e.g., de Haes 2006). Before the method of delivery can be determined, however, the fundamental ethical dilemma needs some resolution: how can we reconcile the conflict between patient values with medical facts and evidence? The debates about SDM and its integration with EBM tend to focus on incorporating the patient’s values as a factor or information within the physician’s evidence-based decision (e.g., Epstein and Street 2011). There is little discussion that conceptualizes the social responsibilities of each person within the clinical encounter and their normative roles within decision-making. My analysis offers a way to move beyond the medical fact/patient values distinction within the ethics of decision-making by drawing on the political sociology of Max Weber whose method of reasoning, called *verstehen*, will provide two alternatives: a way of understanding the conflict between patient values and medical evidence and a solution that reconceptualizes the role of the physician in the clinical encounter.

In the following section, I will demonstrate the forms of rationality that underpin the ethical conflict between patient values in PCC and EBM. Next, I will provide some examples from the field of medicine that aim to improve medical decision-making, including clinical practice guidelines and codes of ethics to demonstrate their forms of rationality. I explain how these guidelines cannot resolve the conflict between facts and values in medicine. Then, I draw on Weber’s political sociology to demonstrate how his method of *verstehen* can be used as lay perspective to integrate PCC into EBM.

## Rationalization and medicine: formal and substantive reasoning

EBM’s solution to integrating patient-values and EBM takes a formally-rational approach. As I will explain in this section, this approach limits the ability of the physician to consider her/his/their responsibility to the patient’s values in the clinical encounter. Rule-guided activity is technical and cannot be concerned with the evaluation of consequences.

Max Weber’s historical analysis of economic activity is helpful for understanding the nature of evidence-based decision-making. Weber’s object of analysis concerned two types of rational social action, which were both oriented to specific goals: *Zweck*- or instrumentally-rational social action is oriented by the auspices of goals and calculated means of attaining them; whereas *wert*- or value-rational social action is oriented by a belief in a value for its own sake, such as ethical or religious beliefs (Weber 1978, 24-25). Weber builds upon this distinction to develop the rationality or mode of reasoning that guides socially organized activity. His work on formal rationality can be used to understand the modern emergence of a particular way of regulating and calculating medical decisions. For example, the goals of medicine are to “maintain life” and “diminish suffering” (Weber [1919] 1946, 144). Formal rationality is characterized by the “degree in which the provision of needs...is capable of being expressed in numerical, calculable terms, and is so expressed” (Weber 1978, 85). Formal rationality aims to secure the ends of socially organized activity through calculable, predictable strategies which are “technically possible and which [are] actually applied” (ibid). Action that is formally rational is expressed in calculable terms with technical interventions, which orient individual actions to the same end.

One of the major contributions of EBM to medical practice is its emphasis on creating clinical practice guidelines (CPGs), which translate the results of medical research into accessible recommendations for clinical practice. CPGs are general guidelines created by medical associations and task forces that evaluate all available medical evidence (i.e., studies about clinical therapies and interventions) in order to produce recommendations for clinicians in their individual practices. CPGs are created to change physician behaviours (Guyatt et al. 2008). EBM aims to eliminate uncertainties surrounding subjective decision-making: if all physicians are following general guidelines about treatment based on the best available research, conventional “expertise” and physician intuition can be replaced by “evidence-based” guidelines (Guyatt et al. 1992; Guyatt et al. 2008).

CPGs are “calculated” inasmuch as they are *both* based upon RCTs and biostatistical calculation *and* prescriptions and procedures that are created with the goal of producing the best possible outcome of any medical decision, be it diagnosis or treatment. For Weber, formal rationality exists when the following can be observed:

*General rules*, which are more or less stable, more or less exhaustive, and which can be learned. Knowledge of these rules represents a special technical expertise which the officials possess. It involves jurisprudence, administrative or business management ... [And] does not entitle the agency to regulate the matter by individual commands given for each case, but only to regulate the matter abstractly. (Weber 1978, 958)

The physician is expected to utilize her/his/their special technical and clinical expertise to execute the recommendations. CPGs are general rules that regulate physician activity abstractly.

It is helpful to briefly discuss the hierarchy of evidence in EBM to further illustrate the principles of formal rationality by which abstract rules and evidence are rendered meaningful in a clinical situation. As noted above, “evidence” is articulated in the assessment of medical research, specifically in randomized controlled trials (RCTs), observational and comparative clinical studies, and mechanistic and expert reasoning. In the theoretical hierarchy of evidence, “any empirical observation constitutes potential evidence, whether systematically collected or not;” this includes both unsystematic observations made by doctors and physiologic experiments (Guyatt et al. 2008, 10). Systematic observations produced by RCTs are considered the best evidence or what medicine refers to as the “gold standard” of evidence. Unsystematic clinical observations that are made by individual clinicians are considered the weakest form of evidence and the most susceptible to the uncertainties of individual sensibilities. Clinician expertise and experience remains an asset for making judgments and decisions in the clinical setting, as mentioned above. Under EBM, physicians are taught to critically appraise the evidence from the medical literature, to order the results they find in scientific studies and determine their worth for the individual patient and her/his/their care.^2^ See Table 1 for an explanation of how EBM ranks the strength of evidence and clinical research upon which judgments can be made.

**Table 1:** Hierarchy of Evidence^3^

**Type of Evidence (Strongest to Weakest)**	**Definition**
**N-of-1 Randomized Controlled Trial (RCT)**	Both clinician and patient are blind to whether the patient is receiving the therapy or placebo. The patient’s symptoms are measured and recorded. Treatment ceases when the clinician decides that the treatment is not effective.
**Systematic Review**	Evaluation and assessment of results from many RCTs on the same therapies or comparisons of more than one therapy for the same illness. The methodological strength and confidence of the findings are evaluated.
**Observational Studies**	The effects of treatment therapies (or no treatment, in some cases) are studied between groups of patients where the clinician has not randomly selected participants.
**Unsystematic Clinical Observations**	Everyday empirical observations from clinicians in their practice.

Ideally, the physician follows the recommendations laid out in CPGs when treating individual patients. Guidelines are created by ranking the evidence and are intended to improve the effectiveness of the physician’s decision in clinical care. These goals are formalized through a set of rules, CPGs that recommend treatment and intervention.

Generalization is another “form of highly abstract rules [which] are formulated and applied” (Weber 1978, 657). In order for formal rationality to be effective, the rules must be regarded as gapless. Other research has addressed the formal rationality of evidence-based medical practice. For example, the work of Timmermans and Berg (2005) demonstrated that EBM played a role in standardizing medical practice. CPGs, they argue, standardize medical practice through generalization: “standards lead to watered-down competition, innovation, autonomy, and creativity, concocting a world of increasing and empty sameness” (19). CPGs inhibit the individual doctor from using alternate modes of reasoning and could shape the doctor-patient relationship as a mechanistic encounter.

Other sociological analyses of the institution of medicine and individual practitioners have found Weber’s work helpful for understanding various aspects of medical reasoning and practice. Following Weber’s work, Hewa and Hetherington (1995) explained why a model of mechanistic reasoning in medicine became predominant by emphasizing the calculability and predictability provided by concrete evidence. Salwitz’s concerns about recommending the operation to his patient is about the confidence he has in the outcome of his decision. He quantifies it: “Hundreds of thousands of patients live active lives with colostomies,” he said, which is a numerical expression of what the outcome of this course of action could be. The doctor uses formal rationality when he recommends the operation to his patient. But, as noted above, the doctor is torn between the values of the patient and evidence, which can be understood by turning to a discussion of substantive rationality.

Formal rationality underlies general rules that are expressed on the basis of quantification, whereas substantive rationality is organized by transcendental values. Weber characterized this form of organization of social action to be substantively rational if actors were motivated by a “criterion...of ultimate values” (Weber 1978, 85). Canadian Codes of Ethics provide an example of substantive rationality in medicine. They are rules for social activity, but they are articulated on the basis of value-rational aims. Ideally, medical action is oriented by a transcendental belief that medicine is a social good. For example, the Canadian Medical Association (CMA) (2004) has stated that physicians have many duties to their patients: these include initiating and dissolving physician-patient relationships, communication, decision-making and consent, privacy and confidentiality, research, as well as duties to society more generally, to the profession, and to oneself. Under the category concerned with “decision-making,” the CMA Code of Ethics states a physician ought to do the following:

Recommend only those diagnostic and therapeutic services that you consider to be beneficial to your patient or to others. If a service is recommended for the benefit of others, as for example in matters of public health, inform your patient of this fact and proceed only with explicit informed consent or where required by law. (2)

Both individual and collective (social) values are central to the physician’s obligations. The Code would recommend that a practitioner recognize and put into motion practices that are committed to social needs (public health) and individual ones. When Salwitz considered what he thought would be beneficial to his patient, he was confronted by the fact that what was “beneficial” could be understood in two senses: one being the calculated outcome, whereas the other about the patient’s values about their quality of life. A Code of Ethics requires a physician to make decisions based on the personal values of the patient, but CPGs rely on formal rationality. Here there is a tension between the two forms of rationality within medical reasoning and practice.

To paraphrase Weber, Codes of Ethics can be conceptualized as an attempt to add a “value rational” dimension to the practice of medicine, which is itself generally an “instrumentally rational” type of action. In *The Protestant Ethic and the Spirit of Capitalism*, Weber analyzed protestant writings in order to understand the relationship between formal and substantive forms of reasoning. He examined how the ascetic lifestyles, and the subsequent obligation to save money inherent in the protestant belief system, rationalized western economic activity. He concluded that the “duty of the individual” to organize her/his/their life according to religious principles (such as saving one’s money) was understood as a supreme good (2002, 16-17). For Weber, values have a moral character insofar as they influence how individuals organize their lives and practices (54). Protestant values, such as asceticism, were “a norm-bound style of life that has crystallized in the guise of an ‘ethic’” (21). Protestants understood and believed in the value of asceticism and organized their lives according to this principle.

The Weberian formulation of “ethics” is interesting sociologically because it allows the Codes of Ethics to be understood as the crystallization of cultural values and beliefs as norm-bound styles of life within a formally rational system of EBM. The ethical obligations laid out in the codes aim to organize medical practice. Under the principles of EBM, practitioners have a duty to make good, evidence-based judgments, and they have a duty to do more good than harm. These duties are the “social ethic” of EBM and signify that the values of medicine have a normative influence on the decisions of individual doctors. To paraphrase Weber, the Codes of Ethics created after EBM imply “a notion of duty that individuals ought to experience, and do, vis-à-vis the content of their…activity. This notion appears regardless of the particular nature of the activity” (2002, 18). Values have a transcendental quality; they represent a collective interest while also affecting the individual’s understanding. In EBM, the formal rationality of medicine has crystallized in the guidelines, whereas the ethical obligations for medical practice are oriented by ultimately substantive rational criteria. Both forms of rationality, however, have taken on a rule-guided form. In order to integrate PCC with EBM, it is important to provide a method that is not simply the technical application of rules to clinical situations but a way to orient decision-making according to both formal and substantive principles.

## Medical ethics and political sociology

Weber’s sociological understanding of ethics emphasizes the physicians’ responsibility to internalize transcendental social values in their practice. The Codes of Ethics fulfill a moral mandate to diminish suffering and maintain life. Now, I turn to a discussion of politics, specifically Weber’s two concepts “the ethic of principled conviction” and the “ethic of responsibility” in order to resolve the disagreement between the formal and substantive forms of rationality that underpin medical practice; doing so will allow me to theorize how these principles can be put into practice at the individual level.

Weber defines politics in an extraordinarily broad definition: “embracing every kind of independent *leadership* activity” (1994, 309). Medicine holds a leadership role over human life by maintaining the health of society. For Weber, political action requires a version of responsibility to which officials, that is, technicians of general rules, are not accountable. In drawing this comparison between politicians and doctors, the tension between formal and substantive rationality can be resolved by adhering to an ethic of responsibility which cannot be bound by formal rationality.

Weber discussed the profession and ethics of politics by drawing a distinction between politicians and officials. Although both roles may be associated with work that is generally considered a part of “political office,” their duties encompass different relations to responsibility:

[The politician’s] actions are subject to quite a different principle of *responsibility*, one diametrically opposed to that of the official. When, despite the arguments advanced by an official, his [sic] superior insists on the execution of an instruction which the official regards as mistaken, the official’s honour consists in being able to carry out that instruction, on the *responsibility* of the man issuing it, conscientiously and precisely in the same way as if it corresponded to his own convictions. Without this supremely ethical discipline and self-denial the whole apparatus would disintegrate. By contrast, the honour of the political leader, that is, of the leading statesman, consists precisely in taking exclusive *personal* responsibility for what he does, responsibility which he cannot refuse or unload on to others. (1994, 330-331)

For an official, these duties are carried out by following the orders of his/her/their superior officers. The emphasis is placed on following instruction rather than on the ends to which any instruction is oriented. Weber might understand evidence-based CPGs as ideally relying on the “discipline and self-denial” of the practitioner who follows the rules^4^. A political leader, however, would have to take responsibility for the consequences that follow from any action, including those of the subordinate officers. The leader must take personal responsibility for what she/he/they decides, and this cannot be deferred to others. Doctors who act with this kind of ethic would understand that the consequences of their decisions are theirs personally, not the creator of the guideline.

The question that arises here concerns the logic of this relationship. Weber does not assume that good judgments are the result of good political leadership, while poor choices are the result of rule-following. Leaders can make poor decisions, too, if they are bound by rule-following: “But is it in fact true that any ethic in the world could establish substantially *identical* commandments applicable to all relationships, whether erotic, business, family or official, to one’s relations with one’s wife, greengrocer, son, competitor, with a friend or an accused man?” (1994, 357). For EBM it is not simply a matter of stating that all good decisions are those that maintain life and diminish suffering.^5^ Doing so would lend itself to creating a body of rules (“commandments”) that ought to be followed and executed in every situation. Codes of Ethics can be conceived as a body of rules that is applicable to all relationships, all patients and their values. But the rules derived from any formally rational system cannot be applicable to every situation, a sentiment which can be found in criticisms of CPGs as well as Code of Ethics (e.g., Goldenberg 2010; Goldman 1980). Further, even proponents of EBM understand that not all uncertainties can be predicted or mastered through rule-following: “Clinicians must be ready to accept and live with uncertainty and to acknowledge that management decisions are often made in the face of relative ignorance of their true impact” (Guyatt et al. 1992, 2421). Although there are bodies of guidelines that aim to control for uncertainty by establishing rules for “application to all relationships,” this statement made by the authors of the original publication of EBM signals that the ethical consequences of judgments matter in medical practice. Ethical orientations can resist rule following through leadership, which goes beyond generalization and application and, instead, consider the responsibility and consequences of one’s actions. This form of responsibility must be accounted for given that the nature of any decision may be in ignorance of its forthcoming consequences. A treatment recommendation, for example, may fail to diminish suffering despite the doctor’s best efforts.

Weber turns to a discussion of consequences and the “ends” to which all action is oriented in order to theorize the ethical responsibilities of political leaders. There are two kinds of ethics for political leaders: the ethic of principled conviction and the ethic of responsibility. First, I discuss the ethic of principled conviction, which is one that follows the adage, justification of the means by the ends:

‘Consequences,’ however, are no concern of absolutist ethics. That is the crucial point. We have understood that ethically-oriented activity can follow two fundamentally different, irreconcilable maxims. It can follow the ‘ethic of principled conviction,’ or the ‘ethic of responsibility.’ It is not that the ethic of conviction is identical with irresponsibility nor that the ethic of responsibility means the absence of principled conviction – there is of course no question of that. But there is a profound opposition between acting by the maxim of the ethic of conviction (putting it in religious terms: ‘The Christian does what is right and places the outcome in God’s hands’), and acting by the maxim of the ethic of responsibility, which means that one must answer for the (foreseeable) consequences of one's actions. (1994, 359-360)

In the original text, Weber enters into a lengthy discussion of pacifism and political leadership. The pacifist refuses to use violence because she is guided by the conviction that violence is never necessary and is inherently wrong. The consequences that may fall from this decision, however, are not the fault of the leader: The leader would always be right because she stuck to her principles. If the outcome was negative, it was “god’s will,” or “wasn’t meant to be,” and so on. If the decision was successful, the pacifist would reaffirm her correct principles. Pacifism is a form of substantive rationality: where decisions are made based on the criterion of ultimate values and violence is always wrong. As Gane (1997) has argued, this form of substantive rationality in politics, “precludes the rational consideration of the consequences of action”; meaning that “conviction overrides all concern for the relation of the means and ends of one’s actions, and that this unconditional commitment precludes personal responsibility for the consequences” (551). To apply this to medical decision-making, an ethic of principled conviction could be observed if the practitioner believed that following the evidence was always the right thing to do, despite patient values, for example. The outcome of the decision would be viewed as separate from the decision itself because the logic follows that all decisions should adhere to the evidence. In other words, the conviction that the principle is always correct supersedes any responsibility for the consequences.

The other form of ethics for political leaders is the ethic of responsibility that understands that “one must answer for the consequences of one’s actions.” Weber described this kind of ethical action as one of maturity, where a leader considers the possible consequences of their action and takes responsibility for it. Weber quotes Martin Luther’s famous “here I stand I can do no other” to demonstrate that the ethic of responsibility means that sometimes in life individuals must “make sins”:

No ethics in the world can get round the fact that the achievement of ‘good’ ends is often tied to the necessity of employing morally dangerous means, and that one must reckon with the likelihood of evil side effects. Nor can any ethic determine how far the ethically good end ‘sanctifies’ the ethically dangerous means and side-effects. The decisive means of politics is the use of violence. It seems that the ethics of conviction is bound to flounder hopelessly on this problem of how the end is to sanctify such a means. Indeed the only position it can logically take is to reject any action that employs morally dangerous means. (1994, 360)

Although Weber is here discussing political leadership, medicine’s mandate to maintain life also deals with matters of both death and life. Rectifying the ends of any medical judgment with the means creates a problematic tension between saving a life and diminishing suffering. Some medical interventions require the patient to endure suffering with the aim of curing cancer, such as Salwitz’s patient’s body being forever altered by the insertion of a colostomy bag in order to increase the likelihood that his cancer would not return (to maintain his life for the longest possible time). As another example, breast cancer treatment sometimes requires a mastectomy, the removal of the breast, thus altering the body and sometimes causing mental and physical suffering (e.g., Griffiths et al. 2010). The work of medicine requires an appreciation of the consequences of any decision, and incorporating patient values in decision-making still requires that physicians take responsibility for the outcomes of their recommendations and judgments.

For Weber, these two ethics are incommensurable: “It is not possible to unite the ethic of conviction with the ethic of responsibility, nor can one issue an ethical decree determining which end shall sanctify which means, if any concession is to be made to this principle” (1994, 362). The ethic of responsibility requires a “slow, strong drilling through hard boards, with a combination of passion and a sense of judgment” (369). For Weber, what makes the ethic of responsibility distinct is its attention to the fact that decisions lack clarity in politics, leading to uncertainty (Barbalet 2000, 339). In medicine, the ends of any medical judgment cannot be rectified with the means because the nature of any action is uncertain (the outcome cannot be known in advance). The desire to save lives or diminish suffering may be the intention, but the unpredictability (and sometimes irreversibility) of medical interventions are limits that cannot be overcome by formally rational rules. Every medical intervention contains this paradox. I will now reformate medical ethics in light of substantive rationality and the ethic of responsibility.

## Verstehen as the method of the ethic of responsibility

In this final section, I will provide an example of how physicians might employ this mode of reasoning in SDM and integrate the principles of EBM with PCC. First I return to the Salwitz example to elucidate further this relationship between doctor and patient and the ethic of responsibility. After putting himself in the patient’s shoes, Salwitz realizes that he may not want to follow the evidence and continues:

I told Stan about my personal “decision.” I told him that although the data was unclear, it was likely he was taking a higher risk of cancer recurrence and death if he did not have the colostomy. I emphasized that a full life was possible with a colostomy. However, I told him that from a standpoint of risk verses perceived quality of life, I might personally choose to avoid the surgery.

Salwitz admits that he changed his judgment from one based on evidence and the outcomes of care (e.g., the likelihood of survival after surgery) to one of “personal decisions.” By shifting from what he calls “Dr. Salwitz” to “Jim Salwitz,” as mentioned earlier, the doctor recognized that decisions can be made on the basis of having a “full life.” Above, the experience of the patient is related to quality of life after the surgery– whether it is *worthwhile* to live, rather than an emphasis on the medical necessity to maintain life. Salwitz’s focus on quality of life harkens back to Weber’s question about whether life is worthwhile living. Medicine (Dr. Salwitz) cannot provide an answer to this question. His reformulation of what it at stake (the quality of life) requires an ethic that deviates from the formal rationality of guidelines, evidence, and the EBM programme. Taking the substantively rational principles of a Code of Ethics to heart also requires making a decision that the physician believes to be beneficial to their patient. Filling in the content of what constitutes as beneficial for the patient would require abandoning a view that only regards the outcome that is most likely to be effective at maintaining life (e.g., based on the evidence). An ethic of responsibility can reserve a place for an alternative way of making medical judgments.

The method by which Salwitz arrived at his conclusions was through a “conversation inside [his] own head.” Weber referred to this method as *Verstehen,* or sympathetic understanding: “Empathic or appreciative accuracy is attained when, through sympathetic participation, we can adequately grasp the emotional context in which the action took place” (1978, 5). The insights Salwitz gained from such reflection were ethical in nature: the questions that the patient must tackle were not only limited by the nature of any human action or decision, but they were questions about the future and the kind of life the patient wanted to have. By recognizing the limits of any medical action or intervention, the physician was able to consider his own preferences as another perspective, a different way of understanding the same problem and choice. This kind of reflection would be marginalized by the formal rationality of CPGs. Verstehen is free from purely rational deicision-making because the ethical relationship between the practitioner and the patient’s understanding of her/his/their life is valued above all else.

By engaging in this method of sympathetic understanding and reflection, Salwitz created a new way of thinking about the patient’s values and the medical recommendations: he described an occasion to rethink the medical recommendations about “what ought to be done” based on the probable outcome of a particular procedure, and he treated it, instead, as a question of the value of a patient’s life. The basis for this method of judgment was not the epistemological commitments of EBM. It was not the probability of the outcome of colon cancer that became the thing by which to formulate the solution. The decision did not become “good” under an overriding conviction to a principle determined by outcomes and probabilities or codes of ethics. Instead, the judgment was based on a commitment to “answering for the consequences of one’s actions.” The impossibility of knowing the consequences in advance required that the grounds for medical decisions were reformulated to include this kind of responsibility. Salwitz stated that he would support whatever decision the patient made: “Stan’s choice is not supported by research, data, my personal experience, nor by experts in the field. Nonetheless, if he decides to choose that path [decline the operation], I will support him” (Salwitz 2012). This decision was made in collaboration between what Salwitz recommends, the information provided by the evidence, and the sympathetic understanding of the patient’s values, his quality of life.

In this example, it is not the outcome of maintaining the patient’s life that provided the overriding principle that organized the medical decision, as that would be a reassignment of the same logic of principled conviction as EBM. Nor do I suggest that evidence, information, and knowledge are irrelevant to any action. Even other critics of EBM have upheld the need for good evidence: “The definition of evidence is important...because it illustrates the struggle between patients, scientists, doctors, and public health administrators over the interpretation of scientific results and how to decide the proper goals of medicine” (Saarni and Gylling 2004, 172). What I argue here is that the principles of a purely evidence-based practice limit the possibility of this kind of ethic of responsibility as an alternative to EBM.

The ethic of principled conviction precludes the possibility of questioning whether the principles to which the rules adhere are right or wrong. Medicine could benefit from a relationship to alternatives such as one formulated here via Weber. Although the Codes of Ethics require that physicians make value judgments, departing from the duties of medical science and the assessment of evidence proposed by EBM, there are limited opportunities to genuinely consider whether maintaining life is a worthwhile objective. It appears in the above example that it was the challenge to Salwitz’s medical authority that incited him to take pause, to exercise his reflection and sympathetic understanding. Further, I do not propose that this ethic of responsibility can be strategically implemented through check-lists on the wards. According to Weber, acting based on transcendental values requires an “intellectual sacrifice” (1946, 155). Salwitz’s judgment cannot be adjudicated as “right” on the basis of evidence, which abandons the formal scientific rationality of EBM. Alternative forms of reasoning, however, can reinvigorate values at the centre of decision-making.

For Weber, the reaffirmation of values in public life requires “courage to clarify one’s own ultimate standpoint” (ibid). Through the method of *verstehen*, the physician’s integrity to patient care can be reconceptualized as the “creative pursuit” of healing in the face of human suffering and illness. The lessons learned from Salwitz is that acting in the best interest of the patient may go against the guidelines, evidence, and mandate of medicine to maintain life and diminish suffering: the patient’s values may lead them to choices that result in their death, and the doctor would not be acting in the patient’s best interest, according to EBM and the Codes, should she/he/they let that happen. PCC, however, requires that physicians learn to sympathize with the patient and the values that guide their choices. A significant amount of the literature in PCC and SDM aims to understand when and why patients want to offer input to the decision about their own care (see, for example, Hajjaj et al. 2010 or Kvale and Bondevik 2008). Verstehen resolves some of those concerns, as the physician can orient their decision-making to the orientation of the patient (cf. Weber 1978, 3): they can empathize about their patient’s motivations and understand their patient’s desires to be involved or not, and learn what the patient values about the *worth* of their life. This form of shared decision-making is both one of communication, which is a major concern of SDM (e.g., Clayman et al. 2012), but one that requires the practitioner to take on the patient’s perspective to gain a personal and accurate understanding of the patient’s desires and values.

## Conclusion

Weber’s work on rationality exposes the tension between formal and substantive forms of reasoning in modern western medicine. His political theory is helpful for resolving the conflicts between the rule-guided action of EBM and leadership required for SDM. The ethical orientation of the ethic of responsibility demonstrates the principle to which medical decision-making can become truly patient-centred, while not being bound by any adherence or conviction to a formally rational system, such as evidence-based CPGs. Weber’s method of verstehen is relevant to medicine insofar as it resolves debates about the role of the patient and their values in shared-decision making, and guides medical judgments by a patient-centred *understanding* rather than merely communication.

More broadly, this paper demonstrates the contribution of Weber’s political sociology to discussions about PCC, which relies on SDM. While there has been debate in the literature about how to apply patient-centredness to EBM through SDM, no one has spelled out this method of thinking for clinician. By using verstehen, physicians can better sympathize and take responsibility for supporting patients’ decisions. It also allows for physicians to consider the values that orient patients’ choices and interpretation of the evidence. While the evidence will be able to tell patients and physicians how to maintain life through the most effective techniques and interventions, the subjective understanding of when one’s life is worth living does not map easily onto EBM’s terrain. Instead, because patient values are so individual, a different mode of reasoning can be employed to try to understand what patients value. This brings me to the second point, that EBM distracts attention away from thinking about the patients’ values because the evidence is prioritized. SDM aims to resolve the imbalance between the evidence and the subjective value of quality of life and how patients view their choices. Verstehen is attuned to both the values of doctors and the objectives of medical practice. By acknowledging that the consequences of medical decisions have to be *lived with* and may not maintain life or diminish suffering, and that these decisions are made by both patients and doctors, dealing with the uncertainty of medical practice can become a collective endeavour.
